# A Complicated Entity: Acute Celiac Artery Dissection with Resultant Pancreatitis, Duodenitis, and Cholecystitis

**DOI:** 10.1155/2020/8453168

**Published:** 2020-01-03

**Authors:** Graham T. Endler, Karleigh R. Curfman, Jason H. Hwang, John L. Gray

**Affiliations:** Duke LifePoint Conemaugh Memorial Medical Center, Johnstown PA 15905, USA

## Abstract

Arterial dissection is a well-recognized pathology often seen in Vascular Surgery offices and Emergency Departments alike; however, visceral arterial dissection is an extremely rare, small subset of this entity. With that, an isolated celiac artery dissection as presented within this report is an exceptionally unique pathology that has scarcely been reported, and due to this, management guidelines are undefined. Given the viscera supplied by the celiac artery, many intra-abdominal structures are at risk for ischemia when damage to the celiac artery occurs, potentially witnessed by this report. Due to the exclusivity of this pathology, we are compelled to report the case of a 71-year-old male who presented with complaints of abdominal pain and was found to have an acute celiac artery dissection, which likely resulted in severe ischemic duodenitis, as well as possibly acute pancreatitis, and questionable influence on cholecystitis.

## 1. Introduction

The purpose of this paper is to report a unique entity and its management that occurred at our facility: acute spontaneous celiac artery dissection with resultant duodenitis, in addition to potentially acute pancreatitis and possibly cholecystitis. True splanchnic artery dissections are a less common clinical entity than aortic, carotid, and other visceral artery dissections; however, they still remain an important vascular pathology that requires appropriate and timely treatment. With continual advancements in imaging technology and endovascular techniques, recognition of these dissections has increased along with the subsequent evolution in their treatment. Celiac artery dissections in the absence of aortic artery dissections are a truly unique pathology, scantly reported in the literature. The recognition of symptoms can be masked, leading to a delay in diagnosis; the clinical picture is often complicated as well due to a lack of recommended treatment guidelines as a result of the infrequency of this entity.

Herein, we present the history of one case involving a 71-year-old male with a previously undiagnosed celiac artery dissection, whose clinical picture was complicated by the development of acute pancreatitis, duodenitis, and cholecystitis. Due to the severe ischemic duodenitis the patient developed, we feel that his celiac artery dissection was significant enough to be the cause of this pathology. In addition, while acknowledging the possibility of a passed choledocholith, given the absence of choledocholithiasis on Endoscopic Retrograde Cholangiopancreatography, we postulate that his acute pancreatitis as well as acute cholecystitis was possibly also sequelae of his celiac artery dissection. Therefore, we provide a review of his hospital course and treatment approach in conjunction with the current literature in regard to diagnosis and management.

## 2. Clinical Course

The patient presented is a 71-year-old male with history significant for Guillain-Barre syndrome and resolved paralysis after plasmapheresis treatments, type 2 diabetes mellitus, and hypertension, who presented with a 4-day history of nausea and intermittent, stabbing epigastric and right upper quadrant (RUQ) pain. He had previously presented to a different facility with similar complaints, for which he was diagnosed with cholelithiasis by ultrasound (US). He denied fevers, chills, acholic stools, and bilirubinuria and was continuing to tolerate intake despite his nausea. He had no prior similar events, no inciting causes, and no worsening or alleviating factors, he denied use of tobacco or alcohol, and his family history was only significant for aortic valvular repair in his father. On physical examination, he experienced RUQ/epigastric tenderness, no jaundice or abdominal distention, and only mild tachycardia (low 100 s). He displayed a leukocytosis (WBC 24.5), transaminitis with hyperbilirubinemia (AST 176, ALT 158, alkaline phosphatase 185, total bilirubin 2.9, direct bilirubin 1.8), and an elevated pancreatic enzyme (lipase > 1100). Abdominal US was significant for cholelithiasis, biliary sludge, and gallbladder wall thickening (1.1 cm), without pericholecystic fluid or ductal dilation. His common bile duct (CBD) measured 7.6 mm.

**Figure 1:**
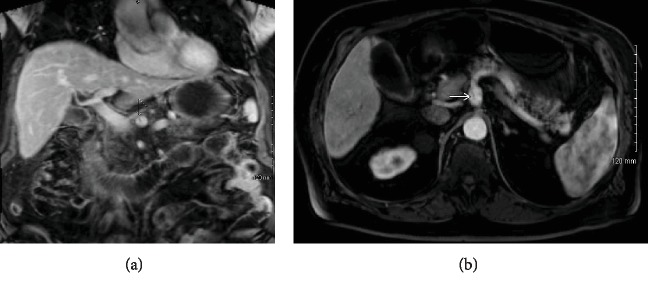
(a, b) Magnetic Resonance Cholangiopancreatography (MRCP) imaging demonstrating signs of pancreatitis and a 1.4 cm celiac artery aneurysm with dissection flap. Given concerns for the possibility of gallstone pancreatitis, the patient underwent MRCP imaging which was the first study to make the diagnosis of a 1.4 cm celiac artery aneurysm and was suggestive of a dissection flap (white arrows). The MRCP was also useful in confirming the diagnosis of acute pancreatitis due to visualized peripancreatic inflammation.

**Figure 2:**
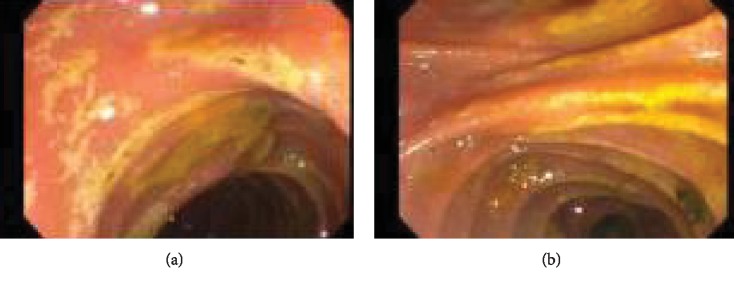
(a, b) Endoscopic Retrograde Cholangiopancreatography (ERCP) showing signs of reported severe duodenitis, likely due to ischemia. After the concern for possible gallstone pancreatitis, the patient underwent an ERCP. With the ERCP, a biliary sphincterotomy and balloon sweep were performed which visualized biliary sludge, but no signs of choledocholithiasis. The ERCP was also suggestive of severe duodenitis, for which ischemic pathology was questioned and, given the patient's known celiac artery dissection, was likely sequelae of the dissection.

The diagnosis of gallstone pancreatitis was presumed and he underwent Magnetic Resonance Cholangiopancreatography (MRCP). The MRCP showed peripancreatic edema and stranding consistent with interstitial pancreatitis and, surprisingly, a 1.4 cm celiac artery aneurysm with filling defect suggestive of focal dissection flap (Figures 1(a) and 1(b)). Given concern for gallstone pancreatitis, he proceeded with Endoscopic Retrograde Cholangiopancreatography (ERCP), for which biliary sphincterotomy and balloon sweep revealed biliary sludge, but no stones. Severe duodenitis was seen on the ERCP, strongly felt to be due to acute ischemia, in correlation to the newly diagnosed celiac artery dissection (Figures 2(a) and 2(b)).

**Figure 3:**
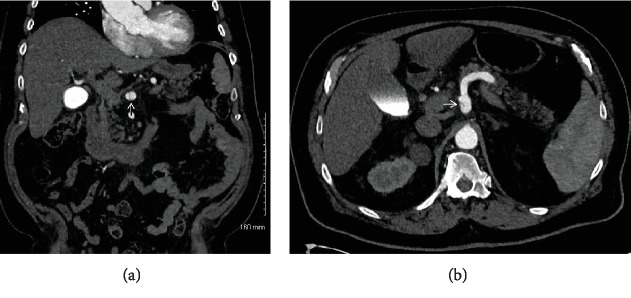
(a, b) Computed tomography angiography (CTA) images illustrating the patient's newly diagnosed celiac artery aneurysm, confirming a dissection flap, and displaying the extent of the dissection flap. CTA imaging was obtained after suspicion was raised on MRCP for a celiac aneurysm and dissection in order to confirm and better characterize the disease. (a) demonstrates the patient's fusiform aneurysm, measuring it at 1.4 cm, as well as displays the celiac artery dissection flap. (b) evaluates the patient's confirmed celiac artery dissection, beginning just proximal to his splenic artery origin and extending to the origin of the common hepatic artery.

As a result of the celiac artery dissection, CT Angiography (CTA) was obtained. This demonstrated fusiform aneurysmal dilation of the proximal celiac artery with a focal dissection flap just proximal to the origin of the splenic artery, which extended to the origins of the common hepatic artery and left hepatic artery (Figures 3(a) and 3(b)). Subsequently, aneurysmal dilation was noted in the ascending aorta (4.6 cm) and left internal iliac artery (1.5 cm). Vascular Surgery was consulted and recommended intravenous anticoagulation and transition to continued oral anticoagulation once stable for discharge, with outpatient follow-up and imaging for monitoring of the dissection and aneurysms.

On hospital day 4, he underwent uncomplicated laparoscopic cholecystectomy as his pancreatitis improved with pain control, hydration, and bowel rest. He tolerated the procedure well and the rest of his hospital course was uneventful, being discharged on day 7.

## 3. Discussion

Arterial dissection has been defined as the cleavage of the arterial wall by blood flow resulting in intramural hematoma between two elastic layers [1, 2]. Isolated arterial dissection, occurring in the absence of aortic dissection, has been a reported entity in the carotid and renal arteries; however, it is rarely reported in the visceral arteries [2]. As per the study produced by Vaidya and Dighe, spontaneous dissection of visceral arteries was first described by Bauersfeld in 1947; however, it reports primarily affecting the renal arteries rather than the celiac artery, as the case presented within [2]. Suspected causes of arterial dissection are numerous and include atherosclerosis, trauma, iatrogenic causes, pregnancy, fibromuscular dysplasia, infectious disease, hypertension, Marfan syndrome, and Ehlers-Danlos syndrome [2]. Spontaneous arterial dissection is more common in males, a reported 5 : 1 occurrence ratio, with an average age of 55 years old [1–3]. Approximately one-half of visceral artery dissections are asymptomatic, though presentation with abdominal pain, weight loss, hemorrhage, and more atypical symptoms like malabsorption, jaundice, or pancreatitis has been reported [4, 5]. Visceral arteries have been shown to have a decrease in dissection rates compared to the aorta and carotid arteries, with dissection of the celiac artery being very rare. As per one study, the first reported case of celiac artery dissection was in 1959, with only 13 cases reported prior to 2001 [3]. Since the advent of improved diagnostic technologies (contrast enhanced computed tomography (CT), CTA, and magnetic resonance angiography (MRA)), the number of reported cases has more than doubled, with Obon-Dent et al. reporting a total of 33 cases in their study from 2012 [3, 6]. Several risk factors described for spontaneous celiac artery dissection are represented in our case; our patient was an older male (71 years), with history of hypertension, and presenting complaint of abdominal pain. Subsequently, he was found to have a left internal iliac and aortic aneurysm, cholecystitis, duodenitis, and pancreatitis.

As described by Wollard and Ammar, pancreatitis can be a rare presentation associated with visceral artery dissection [5]. As such, few cases of reported pancreatitis due to pancreatic ischemia from celiac artery dissection have been published. At the time of production, only four prior cases of acute pancreatitis secondary to celiac artery dissection could be identified after extensive PubMed query. In two separate reports, initial imaging revealed celiac artery dissection, and in conjunction with elevated amylase and lipase, acute pancreatitis was diagnosed. These patients were treated conservatively for pancreatitis with pain control, bowel rest, and hydration as well as blood pressure control, which ultimately alleviated their symptoms without operative intervention [1, 7]. In contrast, one patient with acute pancreatitis and unremitting abdominal pain eventually required an operation. They continued to experience abdominal pain days after presenting and underwent MRA of the abdomen, which visualized a celiac artery dissection. The patient proceeded with selective angiography of the aorta and celiac artery with placement of two stents in the celiac artery, relieving her symptoms [6]. In the publication by Amabile et al., an additional patient with documented pancreatitis secondary to celiac artery dissection was identified and underwent surgical intervention, though details of further management were not described [8]. Although we are unable to prove a direct causation of the celiac artery dissection leading to acute pancreatitis in our case, given the patient's presentation and evidence of severe duodenitis, we hypothesize that pancreatitis was at least aggravated, if not caused by celiac artery dissection, with added evidence of an ERCP negative for evidence of biliary cause.

The contrast in medical and surgical treatment options for celiac artery dissection is striking. Entertainment of procedural intervention should only be employed when the patient is hemodynamically unstable, experiencing persistent symptoms of ischemia, such as abdominal pain, has failed medical therapy for blood pressure control, or if the dissection progresses [2]. Though it has been observed that surgical management should be exercised in those described circumstances, the optimal indicated procedure for celiac artery dissection has not been confirmed and procedures greatly vary in the reported studies. The study by Obon-Dent et al. employed the use of angiography and endovascular techniques to achieve remission of symptoms [6]. Two patients in the Amabile study were identified as having undergone surgical repair. One patient, similar to that of the Obon-Dent report, underwent endovascular repair with the use of covered stent in the celiac trunk, while the other required an upper aortomesenteric venous bypass for combined celiac and superior mesenteric artery dissections; both patients were symptom-free at the time of study production [6, 8]. Furthermore, Glehen et al. described several operative interventions for patients with celiac artery dissection including one patient with resection of the splenic, celiac, and left gastric arteries, another who underwent celiac artery arteriotomy, resection, side to side anastomosis of the hepatic arteries, and reimplantation onto the celiac artery ostium with a graft, and lastly a patient who underwent celiac artery arteriotomy with graft placement between the divided celiac artery and bifurcation of the splenic artery and hepatic artery [9]. Finally, to further contrast surgical management options, in the report by Fenoglio et al., their patient endured operative repair after an increase in dissection size, for which resection of the celiac artery with reconstruction and anastomosis of the gastroduodenal artery and hepatic artery was performed [10]. As described, several operative interventions for celiac artery dissection have been employed, all of which have reported a complete resolution of symptoms by the time of study publication. However, though all of these reports expressed results that were ultimately therapeutic, a concise decision on operative management as the standard of care or its suggested technique has not been agreed upon.

## 4. Conclusion

The case presented in this report is truly unique in pathology. As described, arterial dissection is a known diagnosis that is commonly seen in both the aorta and the carotid arteries, with a decreased presentation in the visceral arteries, especially the celiac artery. Dissection of the celiac artery has scarcely been described in literature, especially prior to the institution of evolved imaging techniques, which are now routinely employed. The pathology of celiac artery dissection in the absence of other vascular dissections is extremely rare, which speaks for the necessity of reporting the case herein. Given that the patient's celiac artery dissection resulted in the diagnosis of severe acute duodenitis and potentially acute pancreatitis and cholecystitis, we feel it is essential to report this case. The occurrence of acute pancreatitis secondary to a celiac artery dissection has only been found in the reported literature on extremely scant occasions, and though we cannot concretely confirm a causal relationship in this instance, the existence is suggested, and thus, it is vital to report details of our patient findings and management for review. It is our hope in the production of this report to provide evidence towards the pathology of celiac artery dissection regarding the presentation, symptoms, imaging, and diagnostic techniques as well as describe previously employed options of various surgical techniques and nonsurgical management for alleviation of symptoms, though a unanimous decision on management technique has yet to be established.
